# Functional analysis and interaction networks of Rboh in poplar under abiotic stress

**DOI:** 10.3389/fpls.2025.1553057

**Published:** 2025-02-26

**Authors:** Jing Wang, Xiaojiao Liu, Yude Kang, Aizhong Liu, Ping Li

**Affiliations:** Key Laboratory for Forest Resource Conservation and Utilization in the Southwest Mountains of China (Ministry of Education), College of Forestry, Southwest Forestry University, Kunming, China

**Keywords:** Rboh, poplar, stress response, protein interaction, CPK

## Abstract

**Introduction:**

Plant respiratory burst oxidase homologs (Rbohs) are essential in the generation of reactive oxygen species (ROS) and play critical roles in plant stress responses. Despite their importance, Rbohs in poplar species remain under-explored, especially in terms of their characteristics and functional diversity across different species within the same genus.

**Methods:**

In this study, we employed bioinformatics methods to identify 62 Rboh genes across five poplar species. We analyzed the gene structure, physical properties, chromosomal distribution, and cis-elements. Additionally, we used qRT-PCR to examine the expression of *PyRbohs* (*Populus yunnanensis Rbohs*) under various stress treatments and yeast two-hybrid (Y2H) assays to confirm interactions with calcium-dependent protein kinases (CPKs).

**Results:**

All identified Rboh genes consistently contained six conserved functional domains and were classified into four distinct groups (I-IV). The number of Rboh members across poplar species was consistent with evolutionary patterns. These Rbohs exhibited relatively conserved amino acid lengths (832-989) and shared basic protein characteristics, including cell membrane localization. Chromosomal distribution analysis revealed an uneven distribution of *PyRbohs* across chromosomes, with abundant collinearity pairs among different plant species, indicating tandem segment duplications and a shared evolutionary origin within group members. Cis-element analysis identified stress-responsive and hormone signaling-related elements. qRT-PCR demonstrated the upregulation of *PyRbohs* under salt, drought, PEG, and ABA treatments. Protein interaction predictions using the STRING database identified potential functional mechanisms of *PyRbohs*, including interactions with CPKs. Y2H assays confirmed the interaction between *PyRbohs* and CPKs, suggesting that CPK binding might regulate *PyRboh* activity and ROS production.

**Discussion:**

Overall, these findings provide a comprehensive understanding of the evolutionary, structural, and functional diversity of poplar Rbohs. They highlight promising candidate genes for enhancing stress tolerance in poplar species and lay a foundation for future research on the molecular mechanisms underlying Rboh-mediated stress responses in poplar.

## 1 Introduction

Biotic and abiotic stresses are major inhibitory factors for plant growth, during which ROS (reactive oxygen species) act as ubiquitous signaling molecules (Mittler et al., 2022). As NADPH (nicotinamide adenine dinucleotide phosphate) oxidases in animals, Rbohs (respiratory burst oxidase homologs) are functional equivalents in plants, and play crucial roles in the generation of ROS in response to hormonal and environmental signals (Baxter et al., 2014; Chapman et al., 2019; Jones et al., 2007). Rboh, located in the plasma membrane, mediates the transfer of electrons from the cytoplasm to the extracellular space, leading to the formation of ROS that provides localized bursts to modulate growth, development, and stress responses (Hu et al., 2020; Baxter et al., 2014; Suzuki et al., 2011). Initially, discovered in human phagocytes during pathogen defense processes that generate ROS, NADPH oxidases consist of six conserved transmembrane domains and a C-terminal region containing both FAD and NADPH hydrophilic domains (Nauseef, 2008). Additionally, they contain two heme groups and two N-terminal Ca^2+^ binding EF-hand motifs, which suggests that NADPH activity is regulated by Ca^2+^ (Kurusu et al., 2015). The human NADPH oxidase protein (NOX) family comprises seven members: NOX1-NOX7 (Martyn et al., 2006). Most Rbohs use NADPH as the electron donor and catalyze the reduction of oxygen to superoxide anion (O^2-^). Subsequently, superoxide anion can undergo either enzymatic or non-enzymatic dismutation to produce hydrogen peroxide (H_2_O_2_). NOX2, the most closely related enzyme to the plant Rboh family, consists of a catalytic subunit composing gp91phox and p22phox, along with three additional cytosolic subunits: p40phox, p47phox, and p67phox (Potocký et al., 2007).

Rboh-mediated ROS production plays a critical role in plant growth, development, and response to abiotic and biotic stresses (Liu et al., 2022; Hafsi et al., 2022; Lee et al., 2022; Angelos and Brandizzi, 2018). In plants, ROS generated by Rboh function as signaling molecules that initiate intracellular signal transduction during various developmental processes. For instance, during the fruit ripening process of pepper, enhanced Rboh activity promotes ROS production and stimulates fruit maturation (Chu-Puga et al., 2019). Rboh can mediate the formation of ROS to manage pollen tube growth of pears (Zhang et al., 2024a). Rbohs of in *Aquilaria* species are involved in metabolism regulation of agarwood (Begum et al., 2023). Additionally, Rboh-mediated ROS facilitate cell wall remodeling, thereby promoting lateral root growth in *Arabidopsis* (Yamauchi et al., 2017). Apart from developmental regulation, Rboh also plays a significant role in plant stress response. For example, Rboh in *Citrus sinensis* is important for plant cold resistance (Zhang et al., 2022). Cotton Rboh exhibits stage- and tissue-specific expression and regulates cotton development under stress conditions by modulating NOX-dependent ROS production (Kobayashi et al., 2007; Raziq et al., 2022; Liu et al., 2020). In strawberries, calcium-dependent protein kinases (FaCDPKs) interact with Rboh to generate H_2_O_2_ under drought stress, thereby activating the plant’s stress response system (Crizel et al., 2020). Similarly, OsCDPK5/11 interacts with RbohH, leading to ROS accumulation under hypoxic conditions in *Oryza sativa* (Yamauchi et al., 2017). In *Solanum tuberosum*, StCDPK4/5 phosphorylates StRboh, activating ROS production in leaves under stress (Kobayashi et al., 2007). Rboh-mediated ROS are believed to play crucial roles in regulating salt stress adaptation in various plant species, including halophytes (Raziq et al., 2022; Liu et al., 2020). The promoter region of *Rboh* in halophytes may contain characteristic sequences involved in chromatin modification, thereby enhancing salt tolerance (Kurusu et al., 2015). For example, the transcription factor bHLH123 activates the expression of *NtRbohE* (NADPH oxidase), thereby improving the salt tolerance of *Nicotiana benthamiana* (Liu et al., 2021). In *Solanum lycopersicum*, the ROS generated under salt stress activate signaling genes such as Rboh, which in turn induce ion homeostasis and osmoregulation (e.g., soluble sugars and proline tolerance) in plants (Raziq et al., 2022).

In addition to participate in plant responses to biotic and abiotic stresses, Rbohs also regulate plant hormone signaling. For example, AtrbohD and AtrbohF are implicated in ROS-dependent ABA (abscisic acid) signaling in *Arabidopsis* (Kwak et al., 2003; Postiglione and Muday, 2020). Rboh isoforms (RBOHH) are associated with ethylene-induced formation of aerenchyma tissue in rice roots (Yamauchi et al., 2017). Furthermore, Rboh dependent signaling and brassinosteroid (BR) signaling interact to regulate cold stress responses in *Citrus sinensis* (Zhang et al., 2022). Furthermore, stress signals activated by Rboh are crucial for plant growth and development. For example, ROS, which are induced by Rboh, mediate plant responses to various abiotic stresses, including ozone, salt, waterlogging, and iron deficiency (He et al., 2017; Joo et al., 2005; Ma et al., 2012; Liu et al., 2017). In addition to Ca²^+^ and electrical signals, ROS also participate in long-distance signal transduction within plants, facilitating intercellular communication (Zhai et al., 2018).

The first Rboh (OsRbohA) in plants was identified in rice (Groom et al., 1996). Since then, Rboh has been found in numerous plant species with varying family numbers. To date, 10 *Rboh* genes have been identified in *Arabidopsis* (Sagi and Fluhr, 2006), 9 in rice (Wang et al., 2013), 7 in grape (Cheng et al., 2013), 14 in tobacco (Yu et al., 2020), 26 in upland cotton (Wang et al., 2020), 7 in strawberry (Zhang et al., 2018), 7 in banana (Zhao and Zou, 2019), 8 in cassava (Huang et al., 2021), 17 in soybean (Liu et al., 2019), 14 in alfalfa (Li et al., 2019a), and 8 in pepper (Zhang et al., 2021). However, the distribution and function of Rboh in *Populus* species remain poorly understood, particularly in *P. yunnanensis*, a commercially valuable poplar species native to southwest China (Xiao et al., 2023). With the aim of enhancing our understanding about the character and function of Rbohs across poplar species, we conducted a comprehensive analysis of Rbohs across five *Populus* species, including *P. yunnanensis.* We identified a total of 62 Rbohs containing conserved Rboh domains from the genomes of the five *Populus* species. Phylogenetic analysis revealed that the Rbohs of *P. yunnanensis* (*PyRbohs*) could be divided into four clusters. The gene structure, domain, and motifs indicated that poplar Rbohs were conserved, with differentiation occurring during species evolution and among groups. Collinearity analysis indicated that segmental duplication events played a key role in the expansion of the *Rboh* gene family. The expression patterns revealed that the Rboh genes exhibit tissue-specific expression and distinct expression patterns under various stress conditions. Furthermore, the function mechanisms of Rboh through protein interaction with other signal proteins in *P. yunnanensis* was elucidated. Our study provides a theoretical foundation for further investigations into the potential functions of Rbohs in *Populus* species.

## 2 Materials and methods

### 2.1 Identification of Rbohs in five poplar species

The reference genome data of *Arabidopsis thaliana*, *Oryza sativa* and *Zea mays* were downloaded from Phytozome (https://phytozome.jgi.doe.gov). The reference genome data of five poplar species (*P. yunnanensis*, *Populus trichocarpa*, *Populus tomentosa*, *Populus alba*, and *Populus euphratica*) were obtained from CNCB (China National Center for Bioinformation, https://ngdc.cncb.ac.cn/) with the accession number PRJCA010101 (Shi et al., 2024). A total of 178 candidate Rbohs were identified in the five poplar species using local BLAST (version: blast-2.12) using 10 *Arabidopsis* Rboh protein sequences as the query, with an E-value of 1e-5. The identified candidate poplar Rbohs were further validated using SMART (http://smart.embl-heidelberg.de/) and the Batch CD-Search Tool (https://www.ncbi.nlm.nih.gov/Structure/bwrpsb/bwrpsb.cgi). The physicochemical properties of the Rbohs were analyzed using Expasy-ProtParam (https://web.expasy.org/protparam/). The subcellular localization of *PyRbohs* was predicted using WOLF PSORT (https://wolfpsort.hgc.jp/). Sequence alignment of *PyRboh* amino acids was performed using ESPript3.0 (https://espript.ibcp.fr/ESPript/cgi-bin/ESPript.cgi).

To validate the predicted subcellular localization of *PyRbohs*, the pCambia1300 plasmid with a GFP label (obtained from Kunming Institute Botany, China Academic of Sciences) was utilized to construct the expression vector. The full-length coding sequences of *PyRboh*s (*Poyun19109, Poyun06279*) were cloned using primers and a high-fidelity amplification enzyme (P505-d1, Phanta^®^ Mix Super-Fidelity DNA Polymerase, Vazyme, Nanjing, China) (**Supplementary Table S1**). The coding sequences were subsequently cloned and inserted into the pCambia1300-GFP vector using the ClonExpress II One Step Cloning Kit (C112-01, Vazyme, Nanjing, China). pCAMBIA1300-35S-PM-mCherry (No. P17271) from Miaoling Biotechnology (China) with cell membrane localization was set as a positive control. *Agrobacterium* (GV3101) cells were used to transfer the recombinant plasmid into *N. benthamiana* leaves, which were cultivated in a greenhouse under a 16 h light (30°C)/8 h dark (25°C) cycle for 30 days. Fluorescent signals were detected using a fluorescence microscope (DM3000, Leica, Wetzlar, Germany).

### 2.2 Phylogenetic, protein and gene structure analysis of poplar Rbohs

The phylogenetic tree of poplar and *Arabidopsis* Rbohs was constructed using MEGA11 (version 11.0.10) with the Neighbor-Joining (NJ) method and 1,000 bootstrap replicates, based on their protein sequences. Motif analysis of poplar Rbohs was performed using MEME (http://web.mit.edu/meme_v4.11.4/share/doc/install.html) with a threshold of 10 motifs. Conserved domains of poplar Rbohs were predicted using the NCBI conserved domain database (https://www.ncbi.nlm.nih.gov/Structure/cdd/wrpsb.cgi). Based on the genome annotation data, gene structures of Rbohs were represented using the Gene Structure View package of TBtools (version v2.142) (Chen et al., 2023). Sequence logos associated with MEME motifs, gene structure, protein motifs, and conserved domains were illustrated using the Gene Structure View package of TBtools.

### 2.3 Chromosome location and collinearity analysis of poplar Rbohs

Chromosome localization of *PyRboh*s was performed using Gene Location Visualize package from TBtools, based on genome annotation data. The collinearity analysis between *P. yunnanensis* and other plants was conducted using the Advanced Circos package of TBtools.

### 2.4 Cis-element analysis of *PyRboh* promoters

To predict the potential cis-elements of *PyRboh*s, we extracted the upstream 2000 bp DNA sequences before the start codons of *PyRboh*s using the GXF Sequences Extract package of TBtools. Subsequently, PlantCARE (https://bioinformatics.psb.ugent.be/webtools/plantcare/html/) was employed to identify the putative cis-elements, which were then visualized using the Basic Biosequence View package of TBtools.

### 2.5 Plant materials and qRT-PCR assays

The cutting materials were collected from *P. yunnanensis* plants that had grown for one year in Kunming (E 102.74°, N 25.17°). Cuttings (approximately 25 cm in length) were cultured for two months in a greenhouse maintained under 16 hours of light at 25°C and 8 hours of darkness at 18°C with natural light intensity and humidity at mixed nutrient medium pots (humus: quartz sand: perlite at 3:1:1) in Southwest Forestry University. For stress treatment, healthy *P. yunnanensis* plants were selected and subjected to the following conditions: drought stress (plants were deprived of water for 2 days, with control plants watered everyday under above culture conditions), ABA treatment (100mL 100 µmol/L abscisic acid (ABA) added in the culture pot for 1 day compared with the same volume water added), salt stress (100mL 150 mM NaCl added in the culture pot for 1 day compared with the same volume water added), and PEG (100mL 10% polyethylene glycol (PEG) added in the culture pot for 1 day compared with the same volume water added), with the methods we did before (Li et al., 2023). After each treatment, at least three biological replicates of plant leaves were immediately collected, frozen in liquid nitrogen, and stored at -80°C for further analysis.

Total RNA was extracted from the stress-treated plant materials using the RNAprep Pure Plant Plus Kit (Cat. DP441, Tiangen, Beijing, China), following the manufacturer’s instructions. 1 µg total RNA was used for reverse transcription with the EasyScript^®^ All-in-One First-Strand cDNA Synthesis SuperMix for qPCR Reagent Kit (AT311-03, Transgene, Beijing, China). The relative expression levels of *PyRboh* genes were determined using gene-specific primers (**Supplementary Table S2**). Real-time quantitative PCR (qRT-PCR) was performed in a 20 µL reaction mixture containing TransStart Green qPCR SuperMix (AQ601-02-V2, Transgene, Beijing, China), using a Bio-Rad CFX96 thermocycler. All experiments were replicated with at least three biological replicates. The results were analyzed using CFX96 software. The real-time quantitative PCR (qRT-PCR) data were analyzed using the 2^-ΔΔCt method, where the ΔΔCt values were calculated relative to the homolog of elongation factor 1 (EF1) in *P. yunnanensis*, which served as the internal reference control. These calculations were performed using the Bio-Rad CFX96 (Li et al., 2023).

### 2.6 Prediction of the interaction proteins of *PyRbohs*

The potential interacting proteins of *PyRbohs* were predicted utilizaing the STRING server (https://string-db.org). All potential interacting proteins of *PyRbohs* were identified based on the predicted interaction relationships in STRING (**Supplementary Table S3**), particularly those predicted from curated databases and experimental data.

### 2.7 Yeast two hybrid assays

Y2H (yeast two-hybrid) assays were employed to validate the predicted protein-protein interaction relationships. Y2H was performed using AH109 strains according to the Matchmaker GAL4 Two-Hybrid System Libraries user manual (PT3247-1, Clontech, USA). The coding sequences of the bait proteins (Poyun00939, Poyun19109) were fused to the GAL4 DNA-binding domain vector (pGBKT7, Clontech, USA), while the coding sequences of the predicted proteins (Poyun270678, Poyun17687, Poyun21683) were fused to the GAL4 activation domain vector (pGADT7, Clontech, USA) (**Supplementary Table S1**). When two vectors were brought into proximity, they activated the yeast reporters. SD/-L-T (SD-Leu-Trp, SD dropout medium with leucine and tryptophan deficiency), SD/-H-L-T (SD-His-Leu-Trp, SD dropout medium with histidine, leucine, and tryptophan deficiency), and SD/-A-H-L-T (SD-Ade-His-Leu-Trp, SD dropout medium with adenine, histidine, leucine, and tryptophan deficiency) medium with different concentrations of aureobasidin A (AbA, 0, 400, and 800 µg/L) were used for screening.

## 3 Results

### 3.1 Identification of Rbohs in five poplar species

To perform genome-wide identification of the *Rboh* gene family in poplar, we used the protein sequences of the ten *Arabidopsis* Rbohs as queries for BLASTP search across five poplar species with a screening threshold of E-value ≤ 0.05. A total of 27, 28, 50, 34, and 39 candidate proteins of the Rboh family were identified in *P. yunnanensis*, *P. trichocarpa*, *P. tomentosa*, *P. alba*, and *P. euphratica*, respectively. Conservative domain analysis was conducted using SMART website and Batch CD-Search Tool, then we identified that all the candidate poplar Rbohs conserved obtained specific Rboh domains including the NADPH, Ferric, FAD, and NAD binding domains, as well as the EF-hand structure. Finally, 9, 10, 18, 13, and 12 Rbohs were obtained in *P. yunnanensis*, *P. trichocarpa*, *P. tomentosa*, *P. alba*, and *P. euphratica*, respectively (**Supplementary Table S4**), which were along with the evolutionary relationship between poplar species (Shi et al., 2024). Total length of the Rbohs varies from 832 to 986 amino acids, with molecular weights ranging from 94 to 112 kDa. All poplar Rbohs were basic, with isoelectric points (pIs) ranging from 9.02 to 9.59 and hydrophilicity values from -0.323 to -0.048. The subcellular localization of poplar Rbohs in the plasma membrane ensured their function, which was verified using transient expression assay (**Table 1**; **Supplementary Figure S1**).

**Table 1:** Protein information of Rbohs in five poplar species.

Species		ID	Amino Acids	Molecular Weight (Da)	PI	Hydrophilicity	Location
*Populus yunnanensis*	1	Poyun19109.t1	948	107660.7	9.42	-0.234	plas
	2	Poyun00939.t1	949	107669.58	9.33	-0.233	plas
	3	Poyun19348.t1	926	104404.03	9.3	-0.25	plas
	4	Poyun30785.t1	909	102756.8	9.21	-0.317	plas
	5	Poyun00667.t1	917	103022.24	9.1	-0.241	plas
	6	Poyun06279.t1	923	105007.39	9.31	-0.279	Plas
	7	Poyun15051.t1	887	101110.96	9.17	-0.293	plas
	8	Poyun06651.t1	846	96770.18	9.33	-0.15	plas
	9	Poyun35235.t1	852	97716.48	9.36	-0.161	plas
*Populus trichocarpa*	1	Potri.001G098300.1	949	107738.68	9.36	-0.235	plas
	2	Potri.003G159800.1.	926	104303.91	9.32	-0.25	plas
	3	Potri.012G111600.1.	909	102997.12	9.15	-0.3	plas
	4	Potri.001G070900.1	926	104102.37	9.1	-0.26	plas
	5	Potri.006G137300.1	926	105425.85	9.29	-0.279	plas
	6	Potri.015G109800.2	909	102783.81	9.21	-0.32	plas
	7	Potri.005G026200.2	887	101082.91	9.17	-0.295	plas
	8	Potri.006G097200.1	846	96712.1	9.33	-0.15	plas
	9	Potri.016G112200.1	852	97739.5	9.41	-0.169	plas
	10	Potri.003G133300.1	948	107578.63	9.41	-0.228	Plas
*Populus alba*	1	XP_034894744.1	949	107835.55	9.23	-0.233	plas
	2	XP_034888226.1	948	107486.51	9.46	-0.238	plas
	3	XP_034921848.1	926	104185.64	9.29	-0.257	plas
	4	XP_034909281.1	909	102709.82	9.17	-0.294	plas
	5	XP_034905438.1	926	104078.37	9.02	-0.269	plas
	6	XP_034905427.1	926	104078.37	9.02	-0.269	plas
	7	XP_034913980.1	926	105359.63	9.23	-0.302	plas
	8	XP_034887704.1	909	102864.04	9.25	-0.312	plas
	9	XP_034912996.1	886	101094.75	9.04	-0.311	plas
	10	XP_034912995.1	887	101181.83	9.04	-0.311	plas
	11	XP_034909280.1	936	105923.52	911	-0.283	plas
	12	XP_034914039.1	846	96306.85	9.23	-0.092	plas
	13	XP_034899498.1	852	97733.5	9.48	-0.172	plas
*Populus tomentosa*	1	KAG6791658.1	949	107835.55	9.23	-0.233	plas
	2	KAG6781122.1	986	112215.28	9.59	-0.223	plas
	3	KAG6782832.1	949	107756.65	9.39	-0.221	plas
	4	KAG6783034.1	926	104207.72	9.28	-0.25	plas
	5	KAG6781322.1	926	104271.81	9.28	-0.261	plas
	6	KAG6753321.1	909	102826.92	9.12	-0.299	plas
	7	KAG6788050.1	926	104018.24	9.09	-0.264	plas
	8	KAG6791419.1	925	104142.33	9.06	-0.29	plas
	9	KAG6754309.1	881	99424.08	9.25	-0.294	plas
	10	KAG6746270.1	939	106318.84	9.18	-0.319	plas
	11	KAG6773184.1	886	101182.89	9.17	-0.317	plas
	12	KAG6769874.1	927	105311.62	9.32	-0.298	plas
	13	KAG6747304.1	900	101979.26	9.25	-0.256	plas
	14	KAG6771648.1	873	99619.45	9.19	-0.098	plas
	15	KAG6744216.1	879	100792.15	9.44	-0.124	plas
	16	KAG6735836.1	879	100781.17	9.48	-0.126	plas
	17	KAG6769568.1	832	94936.27	9.09	-0.048	plas
	18	KAG6771966.1	856	97185.29	9.44	-0.323	plas
*Populus euphratica*	1	XP_011022604.1	948	107733.78	9.48	-0.247	plas
	2	XP_011026969.1	949	108088	9.28	-0.232	plas
	3	XP_011020924.1	926	104341.86	9.31	-0.261	plas
	4	XP_011003531.1	909	102768.81	9.13	-0.296	plas
	5	XP_011015154.1	909	102866.9	9.09	-0.306	plas
	6	XP_011039926.1	926	105391.68	9.22	-0.284	plas
	7	XP_011040313.1	909	102931.97	9.2	-0.318	plas
	8	XP_011036859.1	886	100920.59	9.19	-0.313	plas
	9	XP_011036858.1	886	100920.59	9.19	-0.313	plas
	10	XP_011039925.1	931	105935.44	9.35	-0.257	plas
	11	XP_011019149.1	846	96795.18	9.33	-0.153	plas
	12	XP_011003663.1	853	97627.29	9.42	-0.177	plas

### 3.2 Sequence and functional analysis of poplar Rbohs

**Figure 1:**
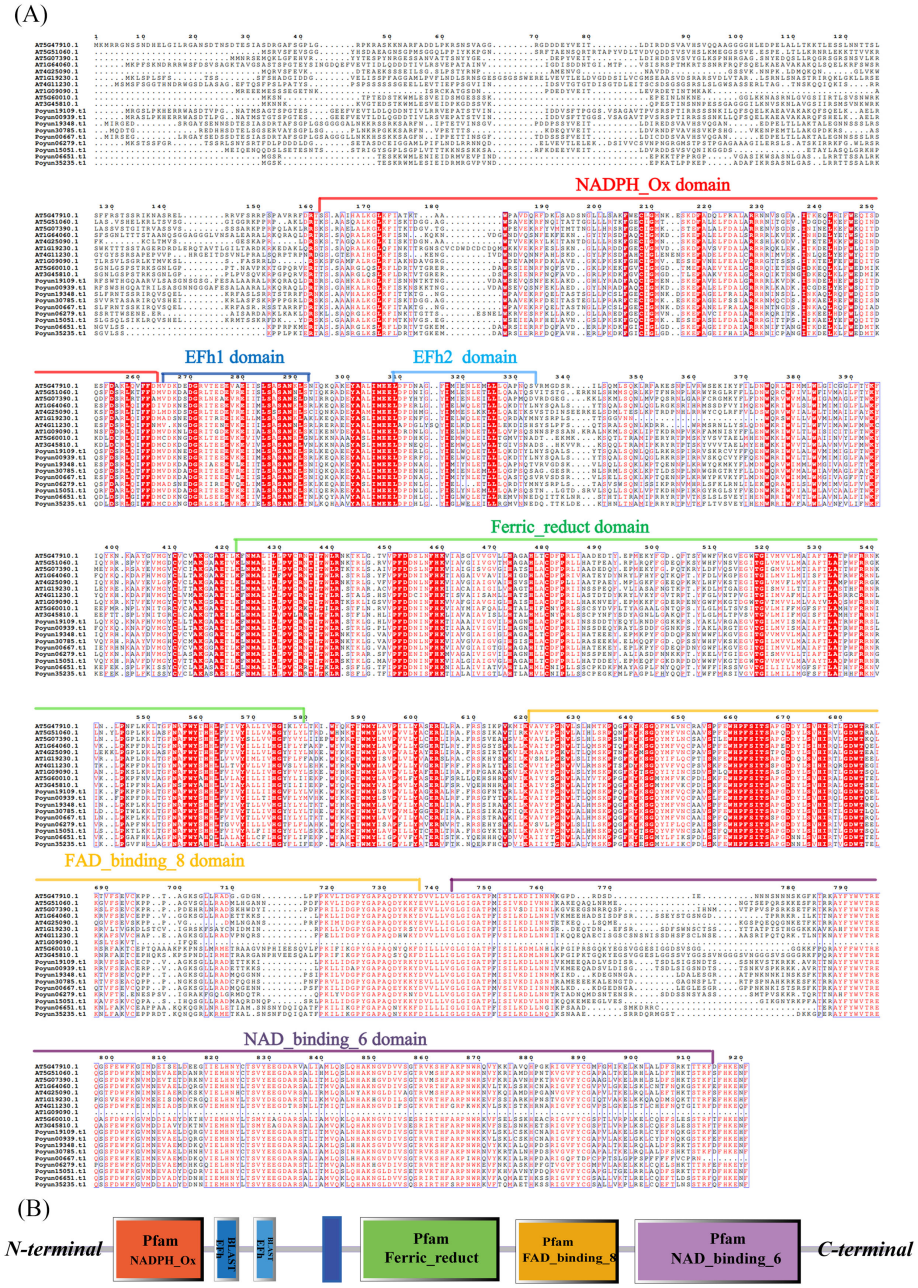
Sequence and structure analysis of *P. yunnanensis* and *Arabidopsis* Rboh proteins. **(A)** Multiple sequence alignment of the protein sequences of AtRboh and *PyRboh* proteins, performed using ESPript 3.0. **(B)** Schematic representation of *PyRboh* proteins, which were presented based on the results of SMART using protein sequences of *PyRboh* proteins, highlighting their respective functional domains. Protein sequences of conserved domains were signed with color frames.

Multiple sequence alignment analysis was conducted with the protein sequence of Rbohs from *P. yunnanensis* and *Arabidopsis*. Following sequence alignment and structural analysis, all nine *PyRbohs* contained specific domains, including the NADPH oxidase, Ferric_reduc, FAD-binding, and NAD-binding (**Figure 1A**) (Potocký et al., 2007). Furthermore, all Rbohs contained Ca^2+^ binding EF-hand structural domain, which was considered pivotal for the functionality of Rbohs. The NADPH oxidase, located at the N-terminal end of the EF-hand motif, was responsible for the generation of ROS in response to plant stress response (Liu et al., 2022). It has been reported that the conserved histidine of Ferric_reduct domain were involved in heme-binding during electron transfer across the cell membrane, facilitated by the NAD- and FAD-binding domains, which were typically found at the C-terminal regions of Rbohs (**Figure 1B**) (Wang et al., 2020). Upon comparison, the *PyRbohs* presented structural characteristics analogous to those in *Arabidopsis*, and their sequences displayed a significant degree of conservation.

### 3.3 Phylogenetic analysis of poplar Rbohs

**Figure 2:**
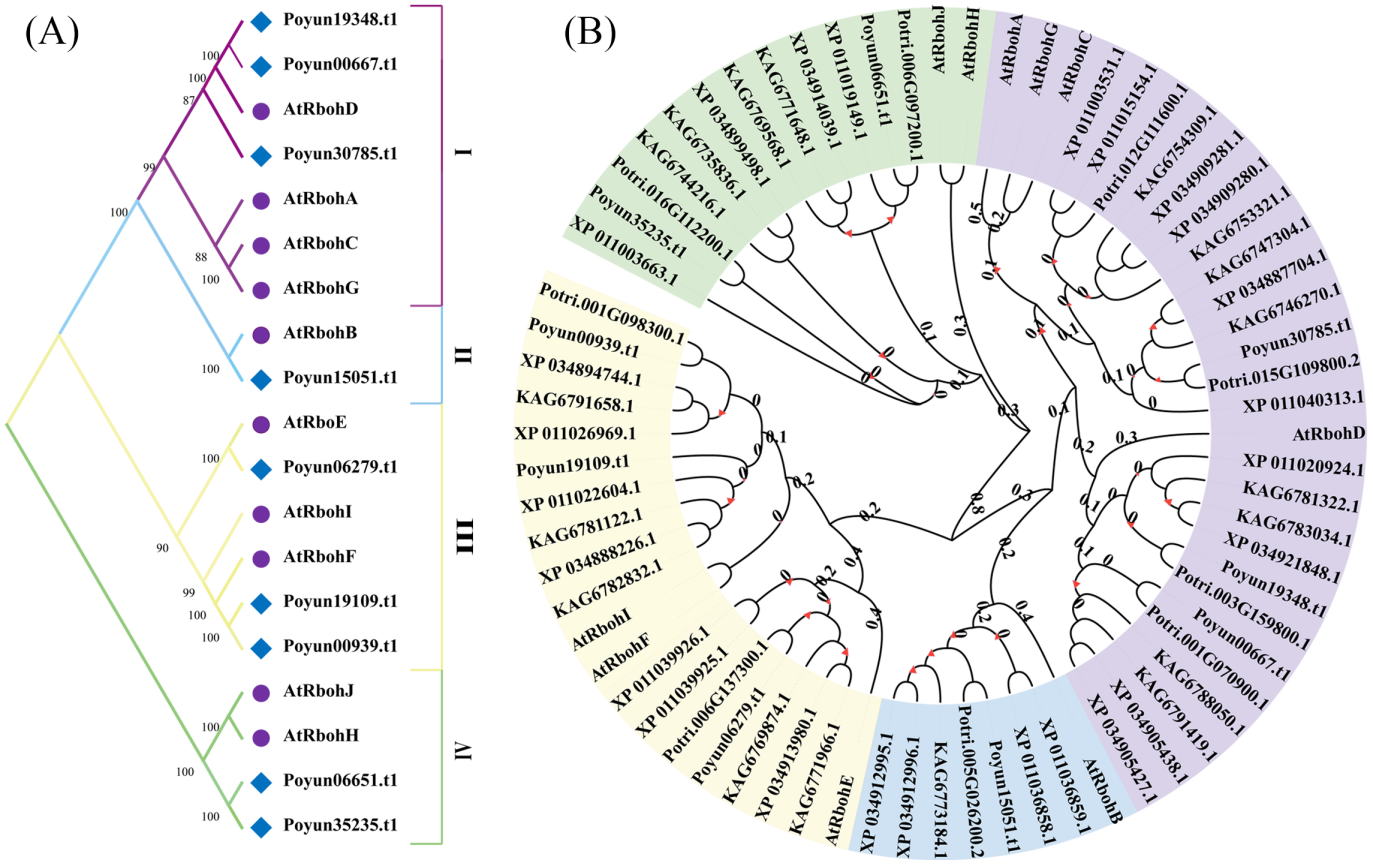
Phylogenetic analysis of poplar Rbohs. **(A)** Phylogenetic tree and classification of *PyRbohs* and AtRbohs. Four distinct groups (I-IV) were identified based on the phylogenetic analysis. **(B)** Phylogenetic tree of 62 poplar Rbohs and AtRbohs. Phylogenetic trees were constructed using the maximum likelihood method in MEGA11, with 1000 bootstrap replicates. Evolutionary branch lengths with bootstrap values greater than 50 were indicated.

To explore the phylogenetic relationships of *PyRbohs*, a phylogenetic tree was constructed using full-length protein sequences of *PyRbohs* and *Arabidopsis* with MEGA11 using the maximum likelihood method and 1000 bootstrap replicates (**Figure 2**). Based on the phylogenetic tree, *PyRbohs* can be classified into four groups, namely, groups I, II, III and IV, which were along with *Arabidopsis*. Groups I and III were the largest, containing the most *PyRbohs* (3 members). Group IV contained two *PyRbohs*, and group II contained one *PyRboh*. All 62 Rbohs from five poplar species could be classified into the same four groups as *P. yunnanensis* and *Arabidopsis* (**Figure 2B**), indicating evolutionary conservation of Rbohs across these plant species.

### 3.4 Protein and gene structure analysis of Rbohs in *P. yunnanensis*

**Figure 3:**
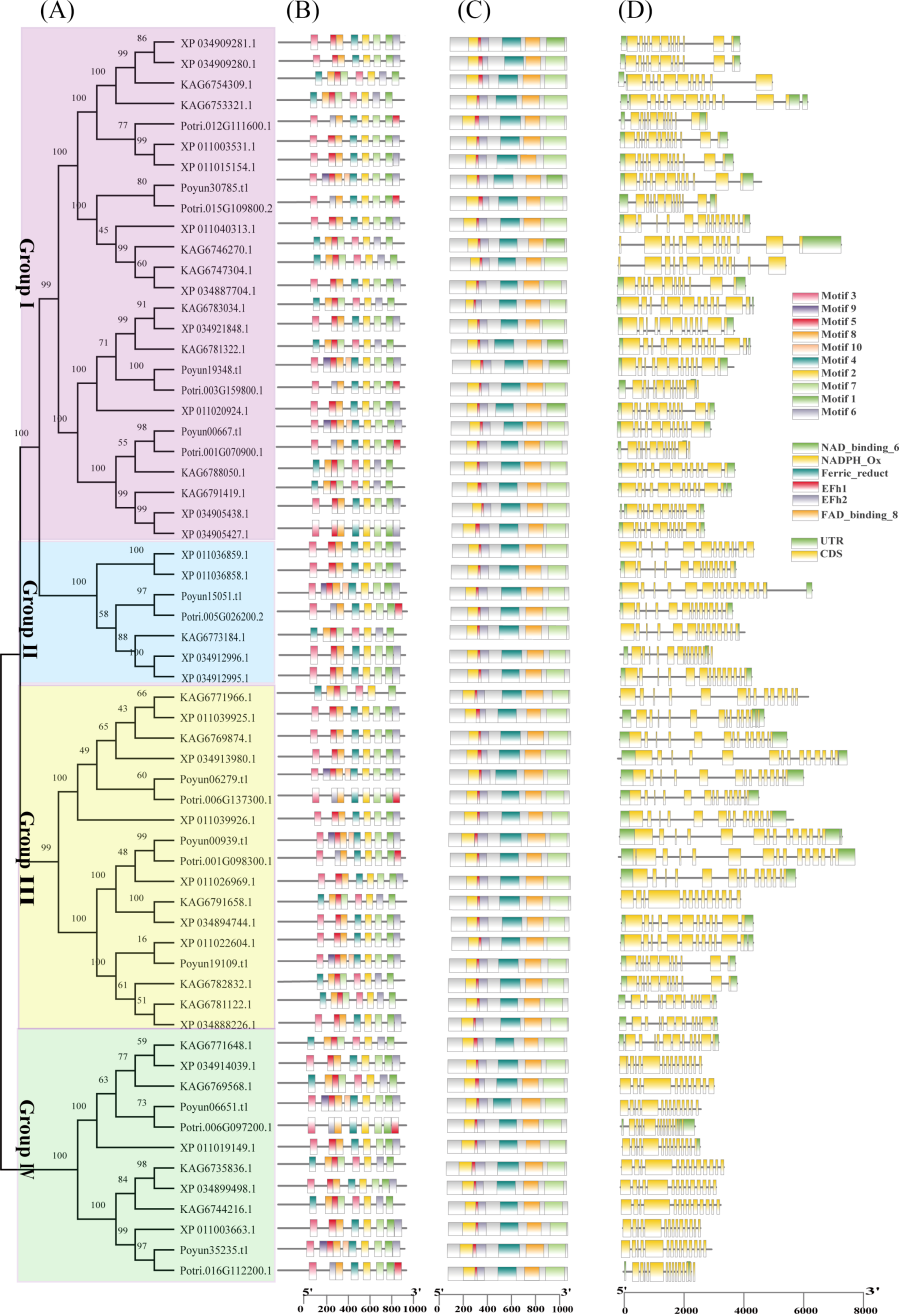
Phylogenetic relationship, protein and gene structure of poplar Rbohs. **(A)** Phylogenetic tree illustrating the evolutionary relationships among poplar Rbohs. The tree was constructed using MEGA11 with the maximum likelihood method and 1,000 bootstrap replicates, based on the protein sequences of 62 poplar Rbohs. **(B)** Analysis of conserved motifs in poplar Rbohs. The motifs were labeled on the right top. **(C)** Examination of conserved domains within poplar Rbohs. Six domains were indicated on the right top. **(D)** Gene structure of poplar *Rboh* genes. Exon and intron boxes were labeled on the right top.

To investigate the characteristics of poplar Rbohs, their protein and gene sequences were analyzed (**Figure 3**). Protein sequence analysis revealed that the same eight to ten conserved motifs were present in all poplar Rbohs (**Figure 3B**). These conserved motifs included an EF-hand at the N-terminus, six transmembrane (TM) structural domains, a NADPH-binding motif and a FAD-binding motif at the C-terminus (**Supplementary Table S5**; **Figure 1**). The conserved domains in poplar Rbohs demonstrated similarity within groups and differentiation between groups (**Figure 3C**). The length of poplar *Rboh* genes varied among different members. A comparison of the gene structures of the poplar *Rboh* genes revealed that the number of exons ranged from 11 to 14 (**Figure 3D**). All poplar Rboh members of groups III contained 13 to 14 exons, but group IV members had shorter lengths. The exon numbers of the groups I and II poplar Rboh genes ranged from 11 to 14. Three *PyRboh* genes of group I exhibited the greatest differences in sequence and gene structure. Typically, within the same evolutionary branch, most poplar *Rboh* genes presented similar exon-intron structures. The protein and domain sequence conservation was also observed among all poplar Rbohs (**Figure 3**).

### 3.5 Localization and interspecies collinearity analysis of *PyRboh*s

**Figure 4:**
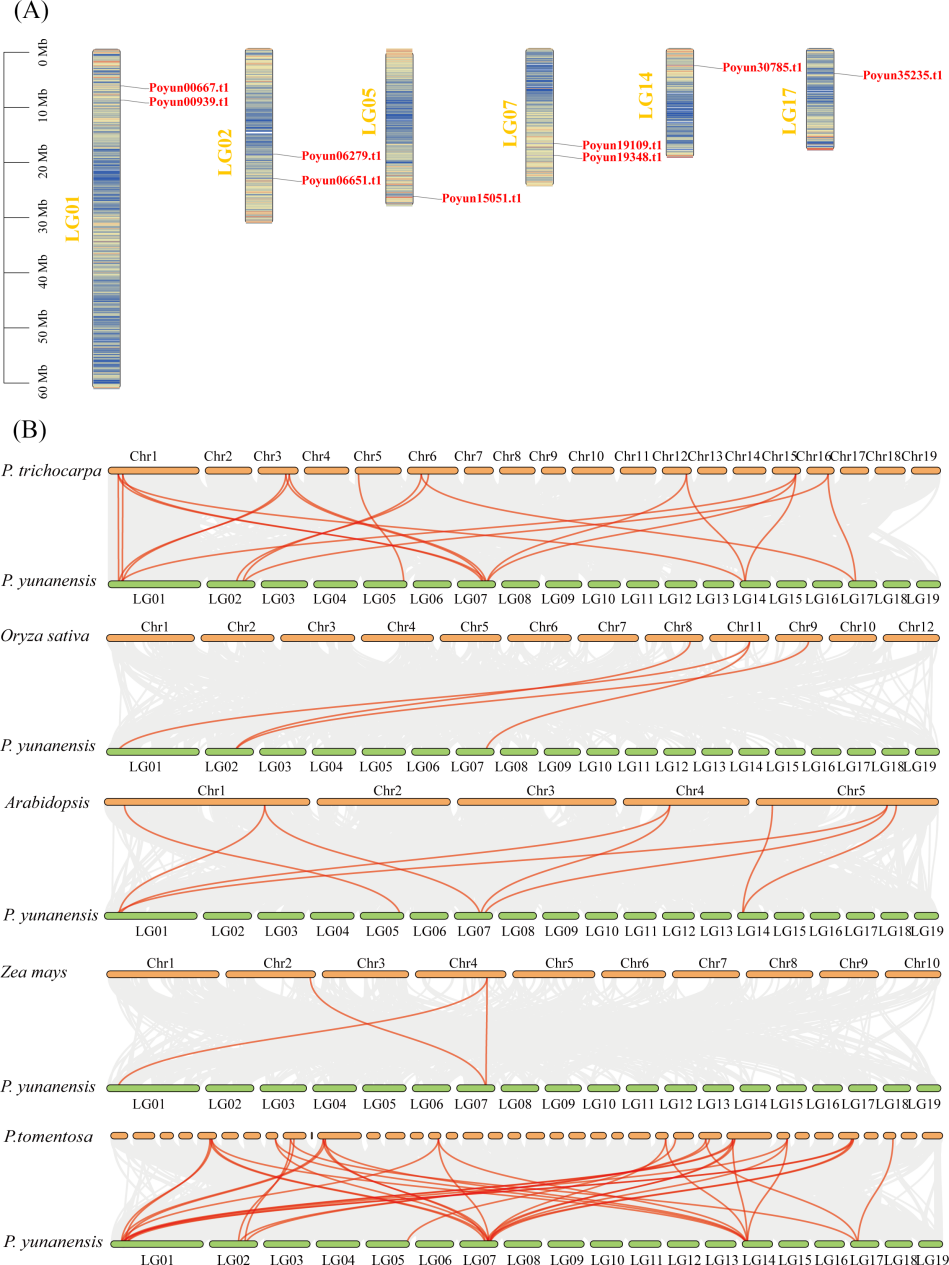
Chromosomal localization and Interspecies collinearity analysis of *PyRboh* genes. **(A)** Chromosomal localization of *PyRboh*s. The chromosome linkage groups (LGs) were indicated on the left of each chromosome. The corresponding genes are labeled on the right. **(B)** Interspecies collinearity analysis of *PyRboh*s genes with those from *Zea mays* (maize), *Arabidopsis*, *Oryza sativa* (rice), *P. tomentosa* and *P. trichocarpa*. Collinearity pairs were connected with red lines. The species and chromosome numbers were indicated near each chromosome for reference.

To investigate the distribution of *PyRboh* genes, their chromosomal was detected (**Figure 4A**). Nine *PyRboh* genes were unevenly distributed on six chromosomes (LGs), namely, LG01, LG02, LG05, LG07, LG14, and LG17. LG01, LG02 and LG07 each contained two *PyRboh* genes. LG05, LG14, and LG17 each contained only one *PyRboh* gene.

To examine the evolutionary relationships, collinearity analyses of *PyRboh* genes were conducted with various poplar species, *Arabidopsis*, rice and maize (**Figure 4B**; **Supplementary Table S6**). Three collinearity pairs were identified among *PyRboh* genes and their homologs in maize, primarily enriched in group I members (*Poyun19348.t1*, *Poyun00667.t1*, and *Poyun19348.t1*). The nine collinearity pairs between *PyRboh* genes and *Arabidopsis* were enriched in the same groups, such as group I members (*Poyun19348.t1*, *Poyun00667.t1*, and *Poyun19348.t1*) and members of groups II (*Poyun15051.t1*) and III (*Poyun00939.t1, Poyun19109.t1*). Four collinearity pairs were identified between *PyRboh* genes and rice, encompassing group I members (*Poyun00667.t1*, *Poyun19348.t1*) and group III members (*Poyun06279.t1*). More collinearity pairs were identified among the other poplar species, and were distributed across all the group members. The collinearity pairs among the same group members revealed the common ancestor origin of the same group *Rboh* genes, suggesting that more gene duplication events occurred within these groups.

### 3.6 Cis-element prediction of *PyRboh*s promoters

**Figure 5:**
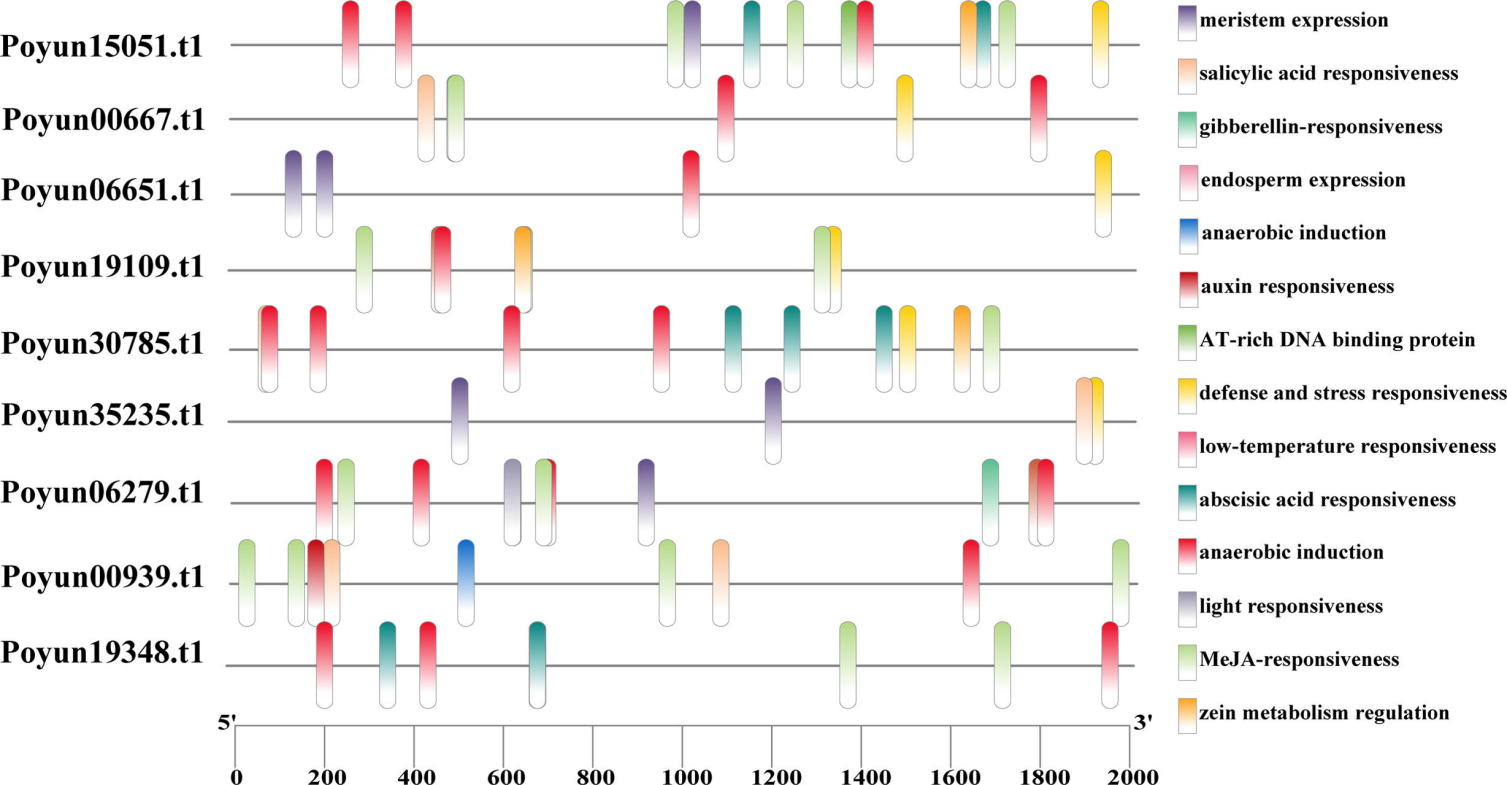
Cis-elements in the promoters of *PyRboh*s. The black lines represent the length of the promoters (2000 bp upstream of the start codon of *PyRboh* genes). The colored rectangles indicate the locations of cis-elements, with different colors representing various cis-elements as labeled on the right.

Cis-elements play crucial roles in the regulation of gene expression (Zhang et al., 2024b). To gain deeper insights into the function and transcriptional regulatory mechanisms of *PyRbohs*, cis-elements within their promoters were predicted within the 2000 bp upstream regions before the translation start site using the PlantCARE database (**Supplementary Table S7**). As depicted in **Figure 5**, a total of 14 cis-elements were identified, comprising stress-responsive elements (defense and stress response, low-temperature response, light response, and anaerobic induction), hormone-responsive elements (ABA, MeJA (methyl jasmonate), GA (gibberellin), auxin, and SA (salicylic acid) response), meristem-related elements, cis-regulatory elements associated with endosperm expression, the binding site of an AT-rich DNA binding protein (ATBP-1), and a maize protein metabolism regulatory element (**Figure 5**). Among these, the auxin response, anaerobic induction, MeJA responsiveness, and salicylic acid responsiveness cis elements were notably enriched within the promoters of the majority of *PyRboh* genes. These findings suggested that *PyRbohs* could potentially have roles under various environmental conditions, contributing to the regulation of poplar growth, development, and multiple stress response pathways.

### 3.7 Relative expression of *PyRboh* under stress conditions

**Figure 6:**
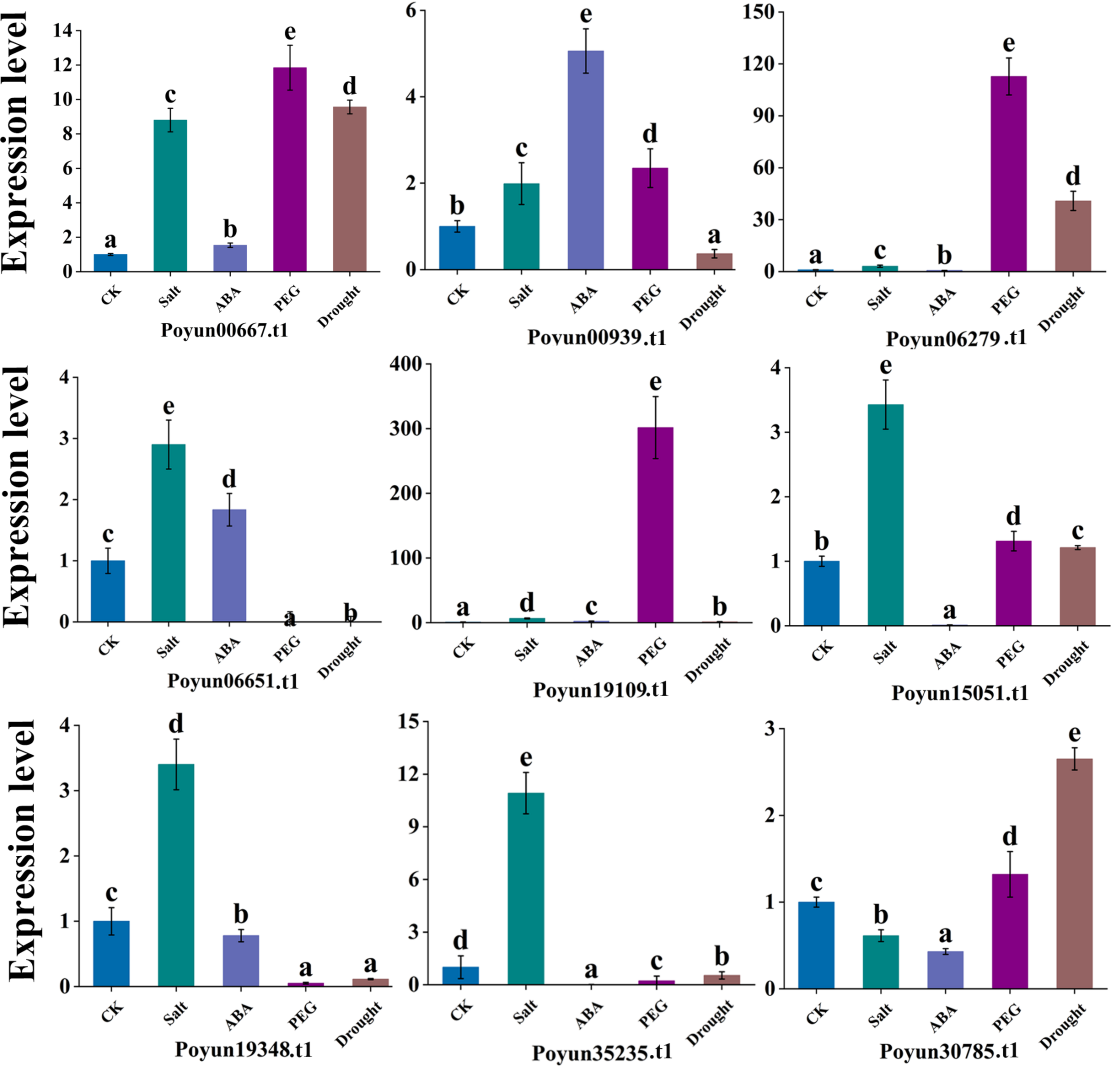
Expression patterns of *PyRboh*s under stress. Expression patterns of *PyRboh*s under different abiotic stress conditions (salt, drought, PEG, and ABA) were analyzed. One-way analysis of variance (ANOVA) followed by Tukey’s test (p < 0.01) was used to determine significant differences. The error bars represent the standard deviation, and different letters indicated highly significant differences in relative expression levels. The experiment was conducted with three biological replicates.

To verify the response of *PyRboh*s to stress conditions, we simulated four different stress conditions (salt, ABA, drought, and PEG) in *P. yunnanensis.* We analyzed the relative expression of nine *PyRboh* genes using qRT-PCR (**Figure 6**; **Supplementary Table S2**). Among these genes, *Poyun19348.t1*, *Poyun15051.t1*, *Poyun06651.t1*, and *Poyun35235.t1* presented significantly increased expression under salt stress, while *Poyun06279.t1*, *Poyun00667.t1*, and *Poyun19109.t1* presented significant up-regulation under PEG treatment. *Poyun00939.t1* exhibited significantly higher expression under ABA treatment compared to other genes, and *Poyun30785.t1* presented the highest expression under drought stress. Except for *Poyun19109.t1*, which showed highly expression under both PEG and salt stress treatments. The results demonstrated that *PyRboh*s exhibited strong response under different stresses, with expression differentiation among the various group members.

### 3.8 Interaction proteins of *PyRbohs*

**Figure 7:**
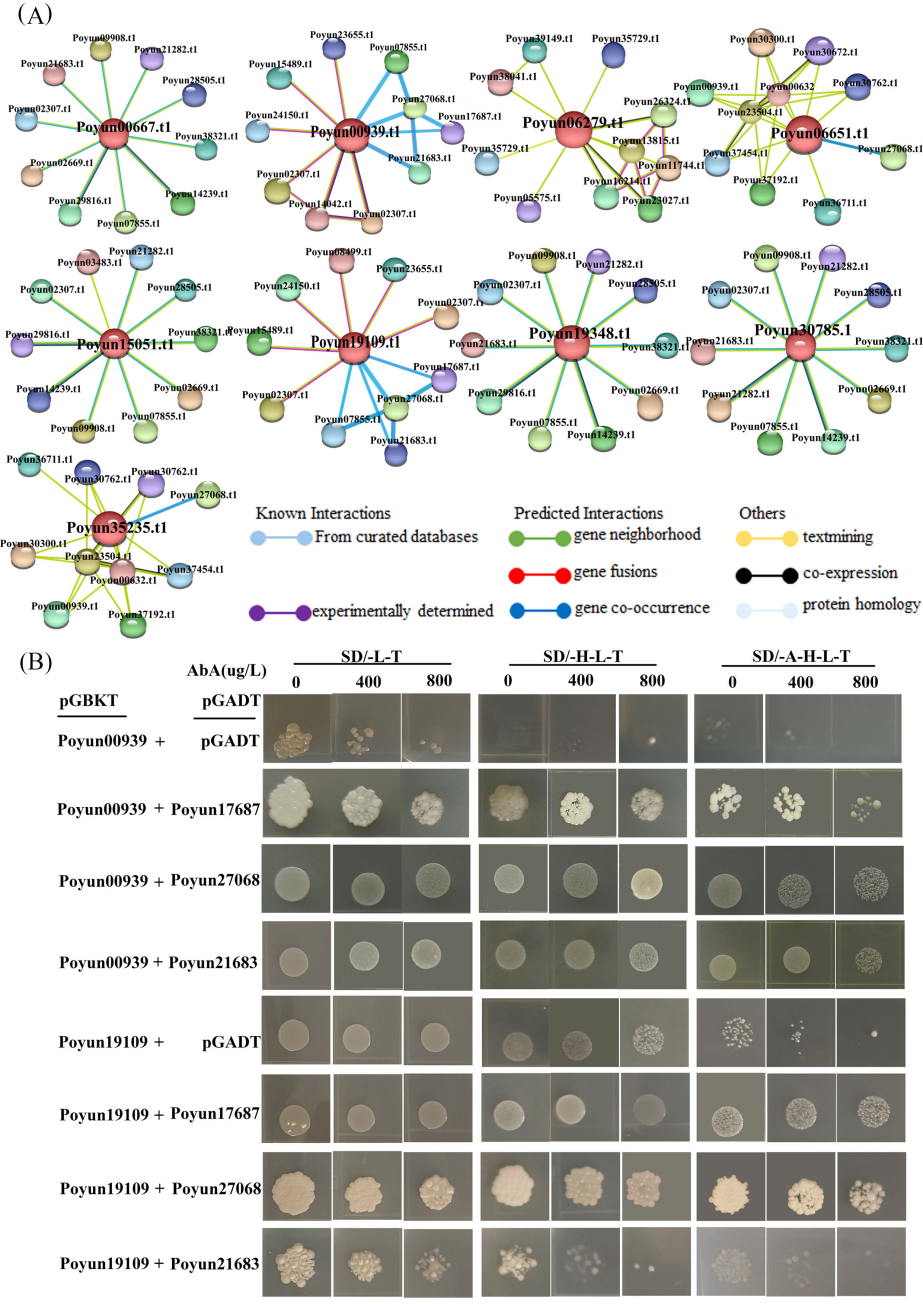
Interaction proteins of *PyRbohs*. **(A)** Predicted network of protein-protein interactions of *PyRbohs* using STRING. Spheres represented interacting proteins, with the orange proteins in the center representing *PyRboh* proteins. The colored lines denoted different types of interaction relationships between proteins: interaction relationships verified by curated databases (blue) or experiments (purple); interaction relationships predicted by gene neighborhood location (green), gene fusion (red), or gene co-occurrence (blue violet); possible interactions of proteins by text mining (olive), co-expression (black), or protein homology (gray-purple). **(B)** Protein-protein interactions detected using Y2H assays. In Y2H assay, the coding sequences of Poyun17687, Poyun27068 and Poyun21683 was ligated to activation domain vectors (AD, as pGADT7-Poyun17687), and coding sequences of Poyun00939 and Poyun19109 was fused to GAL4 DNA-binding domain vectors, (BD, as pGBKT7-Poyun00939). The Y2H yeast strain (AH109) was used in this assay. The pGADT7 without any coding sequences and pGBKT7-Poyun00939, pGBKT7-19109 were used as the negative control of Y2H. SD/-L-T, SD/-H-L-T, and SD/-A-H-L-T represented SD-Leu-Trp, SD-His-Leu-Trp and SD-Ade-His-Leu-Trp medium. Different concentrations of AbA (0, 400, 800 µg/L) were added for screening.

Rbohs are a type of respiratory burst oxidase homologs that can form homodimers and are regulated by their interacting proteins (Kadota et al., 2014). Based on predictions from the STRING website, four *PyRbohs* (Poyun009390.t1, Poyun19109.t1, Poyun06651.t1 and Poyun35235.t1) were predicted to interact with other proteins (**Figure 7A**), especially CPKs (**Supplementary Table S3**). It was known that Rboh activity was regulated by Ca^2+^, suggesting its involvement in signaling pathways where calcium acted as a second messenger (Kurusu et al., 2015). The respiratory burst oxidase homologs (Rbohs) exhibited NOX activity in generating ROS, a process that is activated synergistically with calcium binding to EF-hand motifs and calcium-dependent phosphorylation (Demidchik and Shabala, 2018). To further verify the interactions between calcium-dependent protein kinases and Rbohs, yeast two-hybrid experiments were conducted. The results indicated interactions between *PyRbohs* (Poyun00939 and Poyun19109) and the CPKs (Poyun17687, Poyun27068, Poyun21683) (**Figure 7B**), suggesting their co-regulation in response to stress conditions. The interaction between *PyRboh* and CPK was also verified using the Y2H assay, a popular method for protein interaction (**Figure 7**) (Rao et al., 2014).

## 4 Discussion

Respiratory burst oxidase homologs (Rbohs) are key enzymes that produce ROS in response to hormonal and environmental signals (Hu et al., 2020). Rbohs can provide local ROS bursts to regulate growth, development and stress response (Hu et al., 2020; Baxter et al., 2014), and play crucial roles in plant growth, development and response to environmental stress (Wang et al., 2018). Following the completion of numerous plant genome sequencing projects, *Rboh* gene family members have been identified in various herbaceous and woody plants. However, research on Rbohs in *Populus*, which are widely distributed with highly valued as model plants due to their rapid growth, well-characterized genome, and extensive genomic and transcriptomic resources, remains limited (Demidchik and Shabala, 2018). In this study, a total of 62 Rbohs were identified from five *Populus* species, and their characteristics and functions were analyzed. Our findings indicated that the protein structure of *Populus* Rbohs was conserved and that they could be categorized into four groups, similar to those of *Arabidopsis*, based on their evolutionary relationships. Additionally, gene structure and sequences are conserved within members of the same groups. Chromosome and collinearity analyses of *PyRboh*s revealed a random distribution across six chromosomes and a rich collinearity relationship with other plant species. To elucidate the function of *PyRbohs*, we predicted the cis-elements in their promoters and verified their roles in stress response using qRT-PCR. The function of *PyRbohs* may be related to interacting CPKs, which was verified by a Y2H assay.

Rbohs contain six conserved functional transmembrane domains, which have been used to confirm Rbohs in poplar (Sbarra and Karnovsky, 1959). 9, 10, 18, 13 and12 Rbohs were obtained in *P. yunnanensis*, *P. trichocarpa*, *P. tomentosa*, *P. alba*, and *P. euphratica*, respectively (**Supplementary Table S4**). The number of Rbohs varied among poplar species, corresponding to genome size and evolutionary relationship (Demidchik and Shabala, 2018). Although, the number of Rbohs varied among different plants, the number of poplar Rbohs was similar to that of other woody plants compared with herbaceous plants (Wang et al., 2020; Sagi and Fluhr, 2006; Wang et al., 2013; Cheng et al., 2013; Yu et al., 2020; Zhang et al., 2018; Zhao and Zou, 2019; Huang et al., 2021; Zhang et al., 2021). The protein length and physicochemical characteristics varied among *PyRbohs*, revealing functional differentiation, which was similar to that observed in tobacco and cassava (**Table 1**) (Yu et al., 2020; Huang et al., 2021). Despite differences in physical and chemical properties, the six functional domains were conserved in nine *PyRbohs* and were identical to those in *Arabidopsis* (**Figure 1**) (Huang et al., 2021). The main functional site of Rbohs was the cell membrane, which ensured their role in activating NADH and transferring ion signals (Kobayashi et al., 2006). The subcellular localization of *PyRbohs* was also within the cell membrane, as predicted by online tools and verified through transient transformation experiments (**Supplementary Figure S1**). The Ca^2+^ binding EF-hand structural domains identified in *PyRboh* were crucial for Rboh function (Huang et al., 2021). The NADPH oxidase domain located at the N-terminal before EF-hands, was responsible for generating ROS under stress conditions (Kurusu et al., 2015). Phylogenetic analysis was the primary method for classifying Rbohs, resulting in six groups in *Gossypium hirsutum* L. and five groups in *Aquilaria* species (Begum et al., 2023). Based on the phylogenetic tree of *PyRbohs* and AtRbohs, four groups were identified, which was also conserved in other poplar species, as well as in tobacco and *Arabidopsis* (**Figure 2**) (Yu et al., 2020), indicating the conservation of group classification across poplar species. This classification. The number of poplar Rbohs varied among different groups, with Groups I and II being the greatest, similar to *Arabidopsis* and tobacco (Yu et al., 2020). The conserved protein structure of *PyRbohs* was verified through motif and conserved domain analysis, which was also conserved in all poplar Rbohs (**Figure 3**; **Supplementary Table S5**). These conserved motifs corresponded to the functional domains, similar to those observed in *G. hirsutum* L (Wang et al., 2020). Exon structure may affect gene duplication within gene families (Lambert et al., 2014). The number of exons varied in poplar Rbohs, similar to *G. hirsutum* L. and *Aquilaria* species (Begum et al., 2023), indicating the evolution of Rboh members in poplar (**Figure 3**). *PyRboh*s were unevenly distributed on six chromosomes, independent of chromosome size (**Figure 4**), suggesting segment duplication maybe the reason for gene family formation (Li et al., 2019b). Collinearity analysis provided insights into the evolution of gene families (Gaunt, 2015). Collinearity pairs among *Arabidopsis*, rice and *P. yunnanensis* Rboh members of the same group revealed the evolution and common ancestor of these Rbohs. Compared to other plant species, collinearity pairs of Rboh were more abundant between poplar species, indicating the conservation within the same *Populus* species and the duplication events (**Figure 4**; **Supplementary Table S6**).

To investigate the functional differentiation of different *PyRboh* members, we analyzed their stress response. Cis-elements are key components of most gene promoters and can be binded by regulatory factors to regulate their expression (Chen et al., 2022). Various stress-response cis-elements, including defense and stress response, low-temperature response, light response, and anaerobic induction, were identified on the promoters of *PyRboh*s, revealing the stress-response function of *PyRboh*s (**Figure 5**; **Supplementary Table S7**). In addition to direct stress response elements, hormone-related elements, such as ABA, MeJA, GA, auxin, and SA, have also been identified on *PyRboh* promoters, and played crucial roles in the plant stress response and growth regulation (Waadt et al., 2022). The number and type of cis-elements on the promoters of different *PyRboh*s indicated the functional differentiation of these members, reflecting group different (Kaur and Pati, 2016). To verify the function of *PyRboh*s, qRT-PCR was used to determine the relative expression levels of different *PyRboh*s under stress treatment (**Figure 6**). The expression of *PyRboh*s exhibited group differentiation under various stress treatments. Salt stress was a common response stress for nearly all *PyRboh*s, except *Poyun30785.t1*. PEG and drought, as the predominant osmosis stresses, were the second most significant response stress treatments for *PyRboh*s (Soboleva et al., 2022). ABA, a common hormone involved in plant growth regulation and stress response (Bulgakov et al., 2019), elicited strong responses in four *PyRboh*s. To investigate the mechanisms underlying the function of *PyRbohs*, we predicted the interacting proteins of *PyRbohs*. Interaction proteins can form activated complexes to regulate the expression or activity of proteins (Xu et al., 2023). Several proteins have been reported to interact with Rbohs, including the PRR-associated kinase, BIK1 (Kadota et al., 2014). The co-expression of calcium-dependent protein kinases (CDPK5 and CDPK13) and Rboh can induce ROS production in *N. benthamiana* (Yamauchi et al., 2017), but the mechanisms need to explore. Various proteins were predicted to interact with *PyRbohs*, particularly calcium-dependent protein kinases (CPKs and CIPKs) and the serine/threonine-protein kinase SRK2E (**Figure 7**; **Supplementary Table S3**). The direct interaction between CPK and *PyRbohs* was confirmed using the Y2H assay, providing insights into the stress-response mechanisms of *PyRbohs* (**Figure 7**), which may be activated by calcium signals and involved in stress signal transport and enhance plant stress response. The interaction and functional mechanisms between Rboh and CPK require further investigation. These findings provide valuable insights for investigating the further functions of Rbohs in poplars and offer effective methods for researching gene functions in woody plants.

## 5 Conclusions

This study systematically identified and characterized 62 Rbohs in five poplar species, with a focus on nine *PyRbohs*. Phylogenetic and structural analyses classified all poplar Rbohs into four distinct groups, each exhibiting conserved functional domains. Poplar Rbohs, which were located on the cell membrane, displayed diverse physicochemical properties and gene structures. Analysis of the cis-elements and qRT-PCR confirmed their involvement in stress responses, particularly under drought and salt stress. Y2H assays validated interactions between *PyRbohs* and CPKs, emphasizing their role in regulating reactive oxygen species (ROS) activity. These findings enhance our understanding of how Rbohs specifically contribute to stress tolerance in poplar, by elucidating the molecular mechanisms underlying ROS regulation and stress signal transduction. This knowledge provides a foundation for developing targeted genetic interventions to improve stress tolerance in woody plants, which could have significant practical applications in agriculture and forestry.

## Data Availability

The original contributions presented in the study are included in the article/**Supplementary Material**. Further inquiries can be directed to the corresponding authors.
