# Supplementary figures and images for: Functional analysis and interaction networks of Rboh in poplar under abiotic stress
Source: Front Plant Sci. 2025 Feb 26;16:1553057. doi: 10.3389/fpls.2025.1553057 (PMC11897280; doi:10.3389/fpls.2025.1553057)

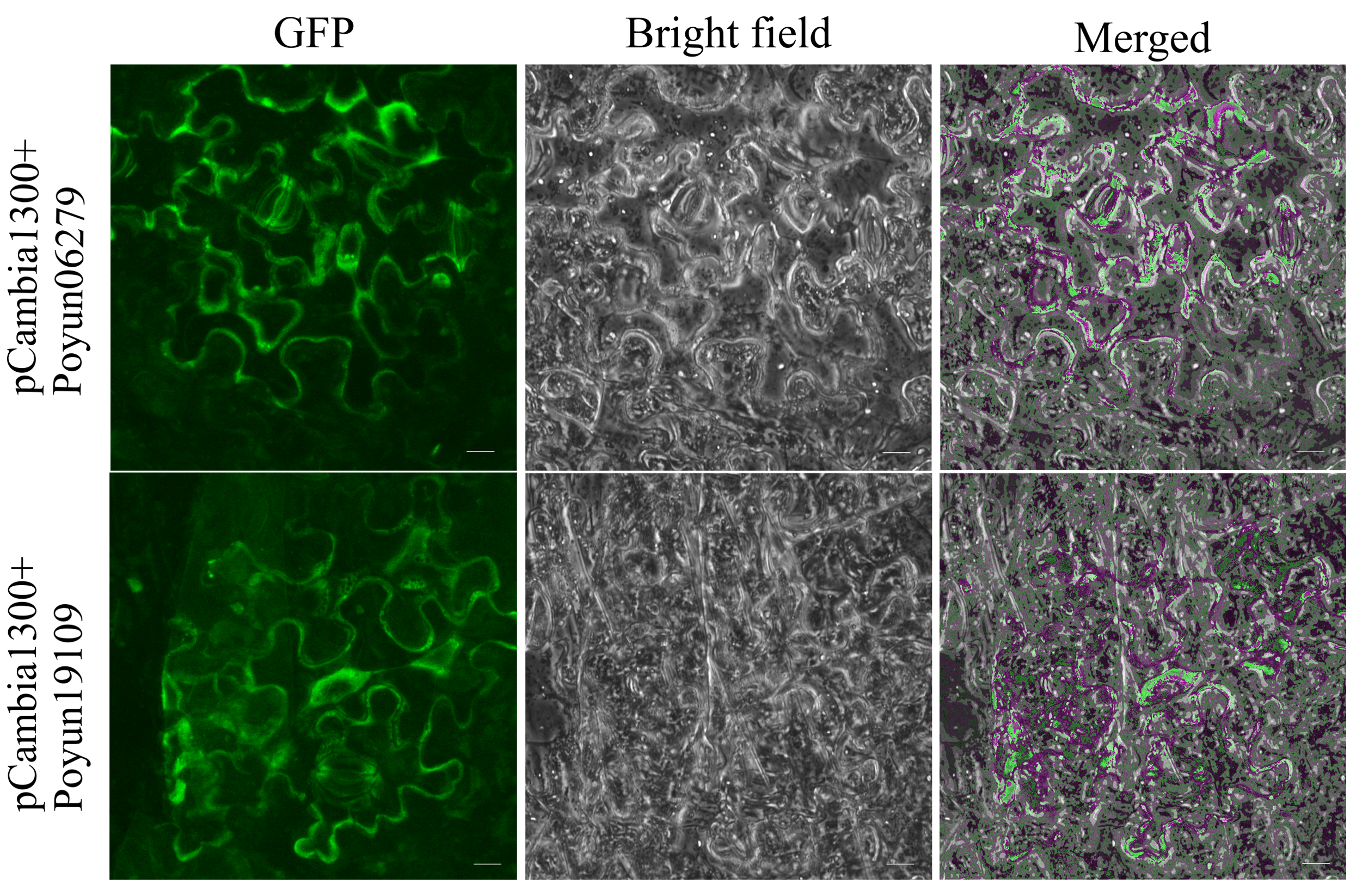

Supplement: Supplementary file 1 [file DataSheet1.zip › Datasheet 1/Figure S1. Subcellular localization of Populus yunnanensis Rboh.tif]
